# Statistical Models Validate Environmental Stability, Body Size and Life History Strategies as Predictors of Maximum Lifespan

**DOI:** 10.3390/ani16152323

**Published:** 2026-07-29

**Authors:** Ioan Sîrbu, Ana Maria Benedek, Andrzej Falniowski

**Affiliations:** 1 Biology and Ecology Research Center, Faculty of Sciences, Lucian Blaga University of Sibiu, 5-7 Rațiu Street, 550012 Sibiu, Romania; ana.benedek@ulbsibiu.ro; 2 Doctoral School of Engineering Sciences and Mathematics, Lucian Blaga University of Sibiu, 10 Victoriei Ave., 550024 Sibiu, Romania; 3 Department of Malacology, Institute of Zoology and Biomedical Research, Jagiellonian University, 30-387 Kraków, Poland

**Keywords:** aquatic and amphibious gastropods, longevity, reproductive strategies, environmental constraints, parametric models, random forests, regression trees

## Abstract

Maximal lifespan is one of the key life history traits, subject to variation and selection, with important implications for evolutionary and ecological strategies. Despite the keen interest in this topic and numerous studies on vertebrate lifespan and its drivers and correlates, there is little research on aquatic gastropods, for which information on life history and ecology is scarce. We used a dataset of 133 species, including the literature data on lifespan (the response variable) and 55 (explanatory) variables covering morphological, life history, ecological and biogeographical characteristics. We applied parametric regression models (generalised linear models, generalised additive models), comparative phylogenetic regression (Phylogenetic Generalised Least Squares) and nonparametric machine learning regression analyses (random forests, regression trees) to examine relationships between longevity and the other variables. We found that both parametric and non-parametric models converged towards similar results. Our findings support life history theory and metabolic scaling predictions, indicating that iteroparous, large-sized species that occupy environmentally stable habitats exhibit extended longevity. Further research should include a wider range of potential drivers to elucidate the underlying mechanisms of longevity and test mechanistic ageing hypotheses.

## Simple Summary

Longevity represents a fundamental life history trait that has been extensively researched in vertebrates. However, the drivers of maximum lifespan in aquatic invertebrates remain insufficiently understood, and our aim was to understand why some species live longer than others. For this, we used aquatic snails as the model group. Aquatic snails are very diverse, with lifespans ranging from less than one year to over 70 years, having different ways of life and inhabiting a wide range of environments. We gathered information on 133 species and compared lifespan by various characteristics, including body size, reproduction, habitat, and geographic distribution. Using several statistical methods, we found mostly consistent results. Species that reach a larger size and live in more stable environments tend to have longer lifespans. Reproductive strategy also matters: species that reproduce multiple times throughout their lives generally live longer than those that invest in a single reproductive event. We also found effects of climate and geographical distribution. These findings support existing theories about how lifespan evolves and what factors shape it. Our models could be used to predict changes in lifespan in the context of global changes.

## 1. Introduction

“An Unresolved Problem of Biology”, the title of the book signed by Medawar [1], still remains relevant after more than seventy years. At first sight, the nearly universal existence of senescence in species of multicellular organisms is paradoxical, given that natural selection supposedly causes the evolutionary trend of increased, not decreased, fitness. As discussed by Comfort [2], many biologists have, therefore, taken the view that senescence reflects an inevitable process of damage accumulation with age, as in complex man-made machines. This, however, does not hold for unicellular organisms, such as bacteria. The large variation in the senescence rates among species also suggests that ageing is subject to variation and selection [2,3,4,5].

In general, two genetic mechanisms of ageing are considered [5]: (1) antagonistic pleiotropy (accumulation of alleles that are beneficial early in life, but deleterious late in life) and (2) mutation accumulation (late-acting deleterious alleles are likely to accumulate in older individuals due to the reduced force of natural selection). Mitochondria also play a central role in ageing through their involvement in energy production, reactive oxygen species (ROS) generation, apoptosis and cellular senescence. As emphasised by Lane [6], the evolutionary origin of mitochondria enabled complex life but also introduced intrinsic bioenergetic constraints that influence lifespan. Variability in mitochondrial efficiency, oxidative stress management and metabolic rate have been proposed as key determinants of longevity across taxa. Finch and Austad [7] proposed two minimal criteria for the lack of senescence: (1) no observable increase in age-specific mortality rate or decrease in reproduction rate after sexual maturity; (2) no observable age-related decline in physiological capacity or disease resistance. Dowling [8] stressed that recent studies report that high rates of extrinsic mortality can lead to the evolution of a longer life, a pattern opposite to that expected under the classic predictions of the evolutionary theory of ageing. Li et al. [5] observed that short-lived species tend to be smaller and have shorter generation and maturation times, while longer-lived species exhibit high rates of iteroparity and a highly concentrated mortality risk (low juvenile survival). This suggests that an extremely long lifespan may emerge from indirect selection on a co-varying trait. There is vast research discussing population genetics and evolutionary models of longevity [9,10,11] and their implementations in the wild [12,13,14,15,16]. Numerous studies concern the molecular basis of ageing and longevity (e.g., [17,18,19,20]). The genetic characteristics, the presence of hybrid and cryptic forms, and the ploidy level of somatic cells may influence lifespan. For example, high ploidy is believed to increase cell resistance to stress, apoptosis (programmed cell death), and genetic failure [21]. However, polyploidy is widely known in plants, which are able to reproduce vegetatively when necessary, but is not typical of animals. The widely known snail with various ploidies is *Potamopyrgus antipodarum* (J. E. Gray, 1843), but it reproduces by parthenogenesis. There are similar examples within some gastropod taxa, but only a few. On the other hand, recently, diploid species have been found whose somatic cells in some organs are polyploid. This influences (enhances) the effectiveness of these organs and thus may positively affect lifespan [22]. However, all these data are rather recent, fragmentary, and not available for most of the species, and there are no data about the possible effect on longevity. Concerning cryptic species, they are common but have escaped morphological determination. Therefore, no data about differences in lifespan between the cryptic species can be found yet, but the increasing use of molecular species identification will address this gap in the future.

The Mollusca are a good object of study of the observed longevity, since their shells (and opercula in many gastropods) may be used for age estimation, and in the fossil material as well [23,24,25,26]. Ponder et al. [27] discuss the techniques (and their limitations) for age estimation based on growth lines, mark-recapture, and isotope ‘sclerochronologies’. It should be stressed that the bivalve ocean quahog, *Arctica islandica* (Linnaeus, 1767), with its recorded lifespan of 507 years [28], is the longest-lived noncolonial animal. In the freshwater pearl shell clam *Margaritifera margaritifera* (Linnaeus, 1758), the maximum lifespan was 210 years in the Arctic [29] and 200 years was recorded for the North American freshwater *Elliptio complanata* ([Lightfoot], 1786) [30]. Other Bivalvia with centenarian lifespans include the fossil *Panopea abrupta* (Conrad, 1849) ([31]; 168 years), *Nordenskjoldia nordenskjoldi* Wilckens, 1910 ([26]; 131 years) and *Cucullea raea* Sharman & E. T. Newton, 1894 from the Eocene Antarctic ([32]; 120 years), the recent North American freshwater *Lampsilis siliquoidea* (Barnes, 1823) ([33]; over 150 years), Pacific mussel *Crenomytilus grayanus* (Dunker, 1853) ([34]; 150 years), Mediterranean and North Atlantic *Glycymeris pilosa* (Linnaeus, 1767) ([35]; 130 years), the widely distributed *Hiatella arctica* (Linnaeus, 1767) ([36]; 126 years) and *Aequiyoldia eightsii* (J. C. Jay, 1839) ([37]; 120 years), geoduck *Panopea generosa* A. Gould, 1850 deeply burying along the North American Pacific coasts ([38]; 120 years), North American Atlantic hard clam *Mercenaria mercenaria* (Linnaeus, 1758) ([39]; 106 years), and Antarctic *Adamussium colbecki* (E. A. Smith, 1902) ([30]; over 100 years).

The extreme longevity reported for several Bivalvia species has prompted numerous studies on the factors that support such longevity [24,32,40]. Extremely long-lived bivalves share life history features: extremely slow and seemingly indeterminate growth [7] in Antarctic, Arctic and Subarctic climates, and the late onset of reproduction, which then continues into old age without a post-reproductive phase [12,41]. In Bivalvia, maximum shell size, development and growth rates are all associated with longevity [39].

In the Gastropoda, the maximum lifespan is shorter. The longest-lived aquatic gastropod is the Antarctic patellogastropod *Nacella concinna* (Strebel, 1908), whose lifespan may reach 60 years [42]. Small buccinid *Ilyanassa obsoleta* (Say, 1822), inhabiting the littoral of the NW Atlantic and parasitised by trematode larvae, may live as long as 70 years, but when not parasitised, it lives only 40 years [43]. *Haliotis cracherodii* Leach, 1814, may live for 51 years [42], *H. rufescens* Swainson, 1822, for 54 years [44], *Aliger gigas* (Linnaeus, 1758) for 40 years [45,46], and several other species for about 30 years. The longevity of the Gastropoda was discussed by Heller [47] and summarised by Ponder et al. [27]. It has to be stressed that the number of aquatic gastropod species for which longevity data are available is quite high, but the data on their biology is usually scarce.

The lifespan in molluscs, similarly to other metazoan groups, is highly variable within species, depending on environmental conditions. European brackish-water *Ecrobia ventrosa* (Montagu, 1803) is a good example: its lifespan in the Mediterranean estuaries is less than a year, 18 months in Danish waters, 15 months in the East Baltic Sea, but as long as 24–30 months in the White Sea [48], indicating that the worse the environmental conditions, the longer the lifespan. This (nearly) general rule also extends to other factors. The boring bivalve *Penitella penita* (Conrad, 1837) from North American Pacific coasts inhabiting soft rocks become mature in 2.5 years, while in the hard rocks they mature at 18 years [49]. Similarly, the boring *Lithophaga patagonica* (A. d’Orbigny, 1846) may live from 4 to 15 years, depending on rock hardness [50]. However, most of the aquatic gastropods—freshwater and marine—are short-lived [27,47], with the life cycle annual or somewhat longer.

The aim of our study was to identify, select and validate potential relations between the maximum lifespan of gastropod taxa and known factors represented by specimen- and population-level descriptors of their life history strategies, environment and biogeography. We focused neither on ranges nor on mean values, but on the maximum lifespan recorded so far for the postlarval stage. Because of the wide variation in the lifespan of the short-lived species and the factors influencing it, in his classic publication, Heller [47] did not even try to present such data; all these species were classified as “short-lived”. Thus, technically, it would be hardly possible to analyse such data. For each studied species, one will obtain only ranges, within less than one to three years, depending mostly on habitat conditions. Therefore, our aim was to study maximal lifespan in long-lived snails, not to study all the possible factors determining the lifespan across the whole range of gastropods, thus trading off generality for feasibility. Considering the knowledge about the life cycles of aquatic gastropods, we assumed the value of three years as a threshold value, excluding all species living shorter. According to this criterion, our database of potential drivers of longevity in aquatic and semiaquatic gastropods comprises 133 long-lived species. It is noteworthy that no species of “Opisthobranchia”, Hygrophila, and fresh/brackish water minute Truncatelloidea are represented, because all these are short-lived. Obviously, the taxon sampling is far from representative or complete. The most easily accessible—shallow water or tidal zone inhabitants, as well as commercially exploited gastropods—are overrepresented in our data. However, one should expect that, even with these limitations, some reliable conclusions about the factors affecting maximum lifespan can be drawn.

Using parametric and machine learning statistical approaches, we identified, tested and validated various predictors of longevity, ranging from body size to evolutionary traits and their environmental requirements. We have learned that lifespan is a multifactorial trait mainly related to morphology, inherited biological features, strategies and responses to environmental conditions.

## 2. Materials and Methods

An extensive literature search resulted in lifespans surpassing three years for 133 aquatic gastropod species. All these data, together with their references, are publicly available in Figshare data repository. The species nomenclature follows the World Register of Marine Species [51] and MolluscaBase [52]. The subclass and order/superfamily classification follows Bouchet et al. [53]. To enable comparative analysis, the data for each species should be complete; thus, a lot of information for some (not numerous) better-studied species has not been included when not available for the others. In the case of habitat, feeding regime and other categorical variables, because species may belong to more than one category, we considered these as binary variables rather than including a single factor, as in the case of taxa. The final selection of 55 variables is presented in Table 1.

**Figure A1:**
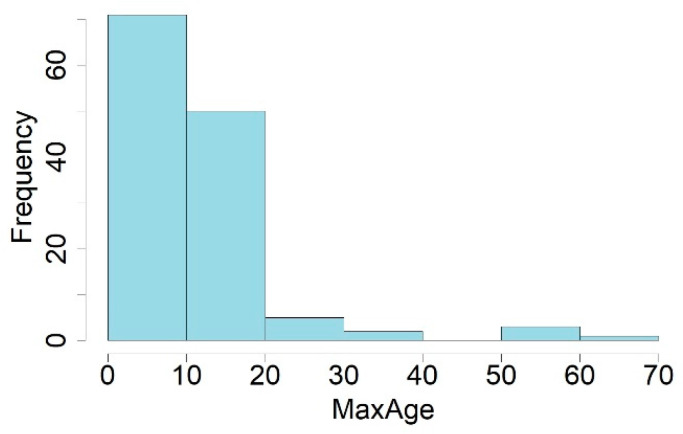
Frequency distribution of longevity (MaxAge, in years) in the species selected for the analyses. Species living less than four years were not included in the dataset.

Data analysis was conducted in R version 4.3.0 [54] and Canoco 5.15 [55]. We used both parametric and non-parametric machine learning approaches. To assess the effect of the species’ traits on their longevity, we used the maximal age (MaxAge) as the response variable. This is a positive, continuous, highly skewed and heteroscedastic variable (Figure A1); therefore, we used the gamma distribution with a logarithmic link function to model it. To account for the potential non-linear effects of the numerical predictors (MaxSize, MinDep and MaxDep), we applied smooth terms in generalised additive models (GAMs), using the function gam in the mgcv package 1.9-4 [56]. In case of categorical variables, smooth terms reduce to coefficients, so GAMs reduce to generalised linear models (GLMs). Thus, we report the results of GAMs for selections of predictors that include numerical variables and GLMs when testing only the effects of categorical variables. Initially, we tested the simple effects of the traits by including each variable in a separate model as the sole predictor. Then we constructed the best model explaining longevity by variable categories, using stepwise forward selection starting from the null model, based on the likelihood chi-square test. To assess the significance of one predictor in addition to those already in the model, we used the lrtest function in the lmtest package 0.9-40 [57]. We selected the set of best models based on the Akaike Information Criterion corrected for small samples (AICc) using the function dredge in the package MuMIn 1.48.19 [58] and reported the coefficients for the averaged model. Unlike in linear models, where the coefficient of determination (R^2^) is the measure of fit, expressing the variance of the response variable explained by the predictors, in generalised models (GLM and GAM) the R^2^ is not appropriate. Instead, there are other measures (pseudo-R^2^) of model fit, and we chose the explained deviance. Deviance measures how much the fit of the models deviates from that of the saturated (perfectly fitting) model, and the explained deviance expresses how much of the improvement over the null model is accounted for by the predictors.

To partition the explained deviance among categories of predictors, we used the function gam.hp in the gam.hap package 0.0-5 [59]. To account for potential differences among gastropod lineages in the relationship between longevity and species traits, we constructed generalised linear mixed models (GLMMs) with a gamma distribution, using the function glmer in the package lme4 2.0-6 [60]. To avoid model overfitting, we evaluated multicollinearity of predictors by calculating the variance inflation factor (VIF) using the function check_collinearity in the package performance 0.17.1 [61].

Among machine learning methods, we used random forests (RFs) in the package randomForest 4.7-1.2 [62] and regression trees (RTs) in the package rpart 4.1.27 [63]. In addition to ranking variables’ importance, RFs add nonlinear modelling, interaction detection and robustness to collinearity, but they do not test the significance of the predictors. RTs identify threshold effects while revealing the interaction structure. In the RF regression, we ranked the importance of variables in the prediction of lifespan by their Percent Increase in Mean Squared Error (%IncMSE), which quantifies how much the prediction error increases when a variable is permuted. The higher the value, the more important the predictor is.

To evaluate and visualise relationships among predictors and among the analysed species, we used Principal Components Analysis (PCA) on centred and standardised values. For clarity reasons, we illustrated only the best-fitting 40 predictors and species.

To test the robustness of our results, we conducted sensitivity analyses, both threshold-based and phylogenetic. Because the maximum lifespan threshold of three years in our original dataset may affect the estimated relationships, we performed a sensitivity analysis using alternative lifespan cutoffs: four, five and six years.

**Figure 1:**
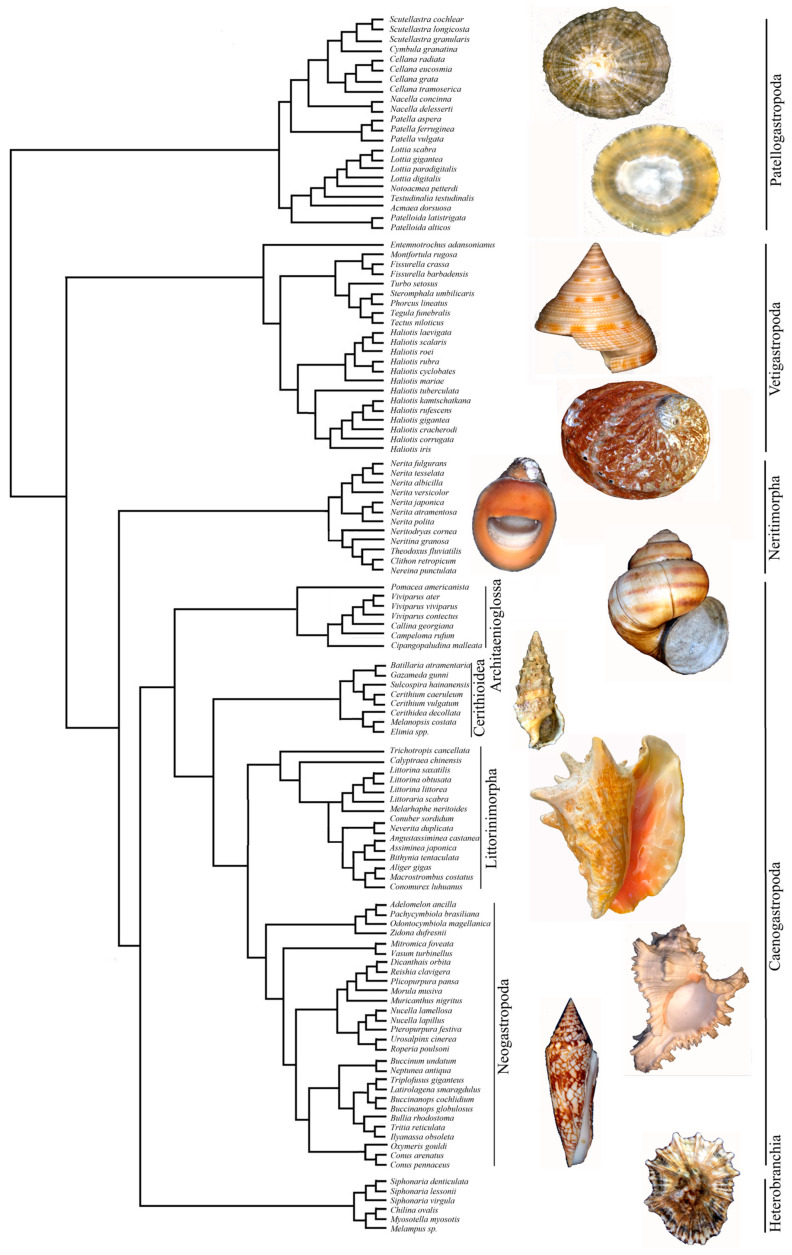
Phylogenetic tree of aquatic gastropods used in the evolutionary comparative analyses.

Since no complete molecular phylogeny is available for all species in our dataset, and there are either complete genomes, complete mitochondrial genomes, or various loci only, and sometimes even morphology-based phylogeny is available for some genera, we compiled partial trees from the literature [64,65,66,67,68,69,70,71,72,73,74,75,76,77], with unresolved or missing taxa grafted at the lowest reliably resolved taxonomic level (Figure 1). We evaluated the role of phylogeny as a driver of longevity by performing a phylogenetic autoregression and tested it using Moran’s I autocorrelation [78] in the package ape 5.8-1 [79]. To assess the effect of shared ancestry, we constructed simple models (with one predictor) within the comparable phylogenetic regression framework, using Phylogenetic Generalised Least Squares (PGLS) with estimated Pagel’s λ in the package caper 1.0.4 [80]. These models do not account for non-Gaussian distribution; therefore, we log-transformed the response variable. Because PGLS requires branch lengths, we used Grafen’s method, which assigns branch lengths based on the topology [81], considered a more reasonable approximation for comparative analyses than simply setting every branch length equal.

## 3. Results

### 3.1. Response of Longevity to Individual Species Traits

The lifespan of gastropod species included in our data set ranged between 3 and 70 years (species living less than 3 years were not included), with most species (53.3%) living up to 10 years, whereas only 9% lived longer than 20 years (Figure A1).

**Figure 2:**
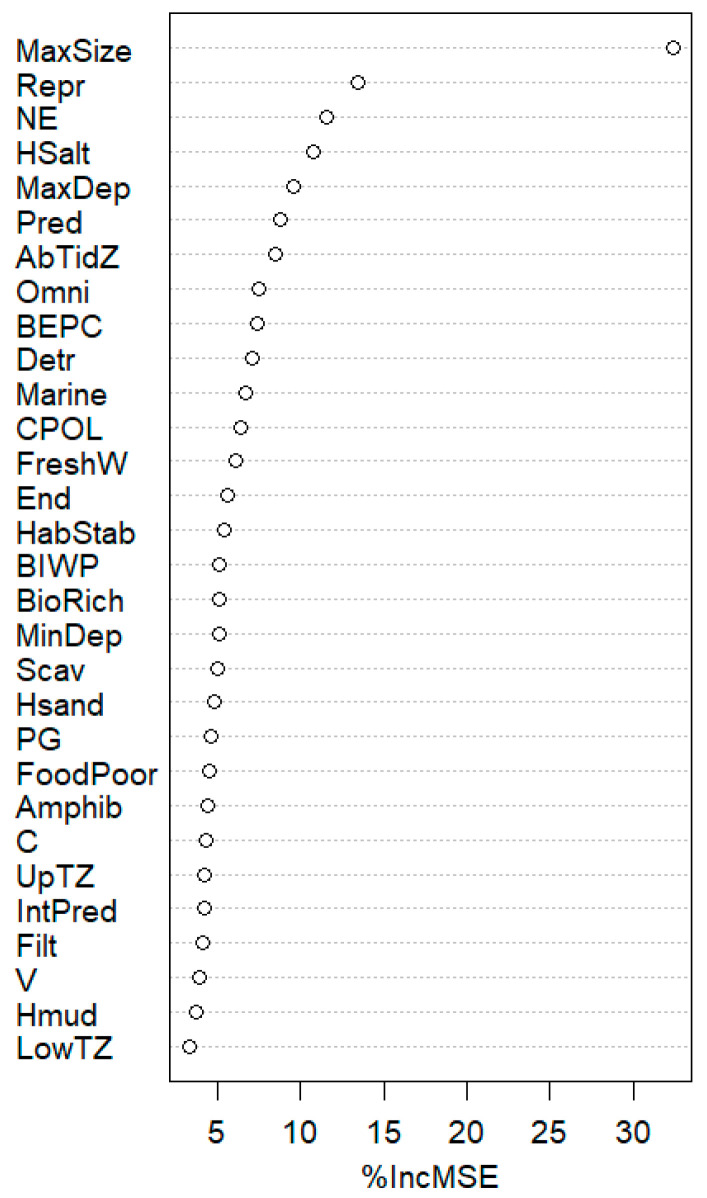
Ranking of the most important 30 independent variables according to their decreasing value of Percent Increase in Mean Squared Error (%IncMSE) by random forest regression. Codes of predictors are given in Table 1.

In the RF regression, body size (MaxSize) was the most important predictor, followed at a lower order of magnitude by reproduction strategy (Repr), taxonomic order (Ord), salt marsh habitats (HSalt), maximal depth (MaxDep) and others (Figure 2).

**Figure 3:**
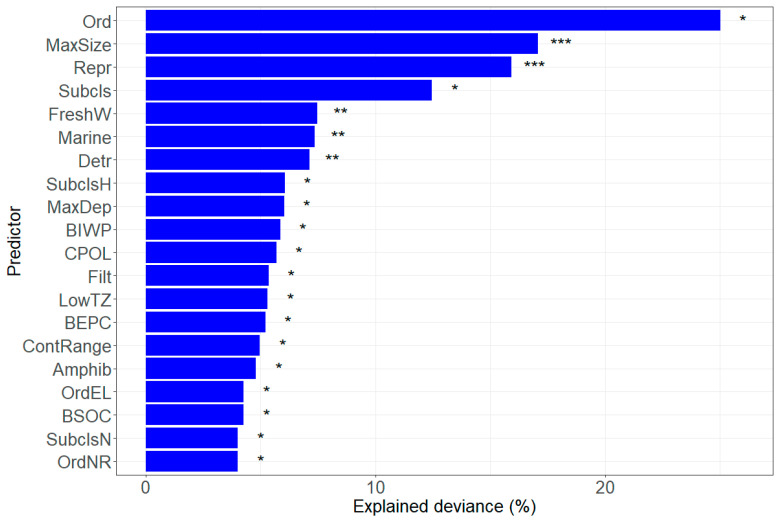
Simple effect (in descending order) of variables on the longevity of gastropod species expressed in terms of explained deviance and their level of significance: ***—*p* < 0.001, **—0.001 < *p* < 0.01, *—0.01 < *p* < 0.05. Only predictors with a significant effect are illustrated. In generalised linear models the explained deviance is one of the equivalents of R^2^ from linear models. Codes of predictors are explained in Table 1.

Using GLM, we evaluated the simple effects of all predictors considered in our study. Overall, the effect of taxa was significant (*p* < 0.02), with order (Ord) having the highest explained deviance (25%) and subclass (Subcls) having about half (Figure 3, Table A1). However, when considering the deviance per degree of freedom, the values are small (2.27% and 3.16%). Thus, when considering each order or subclass as an independent predictor, most effects were not significant. Only snails in subclasses Heterobranchia and Neritimorpha and orders Ellobiida and Neritoidea were found to be significantly shorter-lived than the others. Among the other predictors, maximum size (MaxSize) and reproduction strategy (Repr) had the strongest and most significant effect (*p* < 0.001); longevity increasing with body size and iteroparity (Table A1). Other significant predictors, having an explained deviance lower than 8%, reflected environment: FreshW—freshwater inhabitant, Amphib—amphibiotic, Marine—marine, LowTZ—in lower tidal zone, and MaxDep—maximum depth. Feeding strategies: Detr—detritus feeder, and Filt—filtrator. Biogeography: BIWP—Indo-West Pacific, CPOL—polar climate, BEPC—Eastern Pacific Ocean, BSOC—Southern Ocean and ContR—continuous range. Deeper waters, colder climates and larger geographic ranges were associated with increased longevity (they had positive coefficients; see Table A1).

### 3.2. Relationships Among Predictors and Among Species

**Figure 4:**
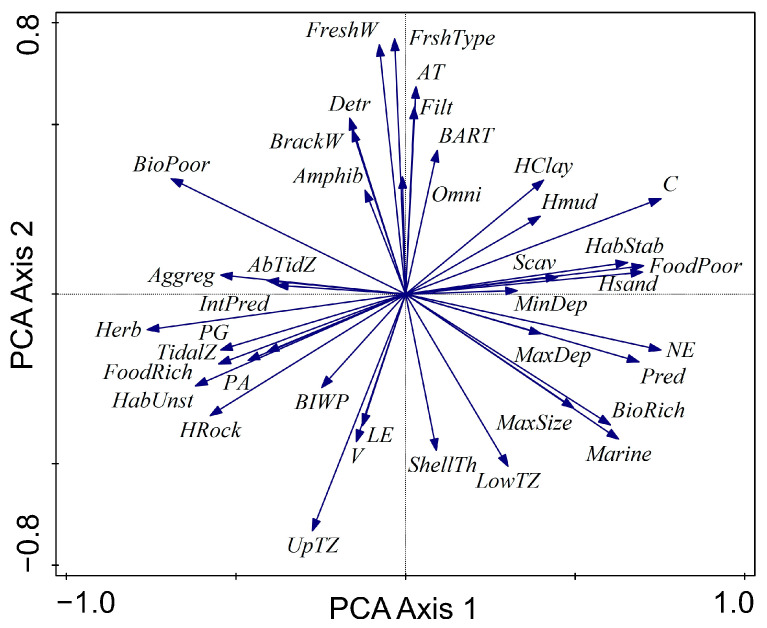
Ordination diagram for the Principal Components Analysis (PCA) of 40 predictors selected based on best fit. Only the first two PCA axes are represented. Codes of predictors are given in Table 1.

**Figure 5:**
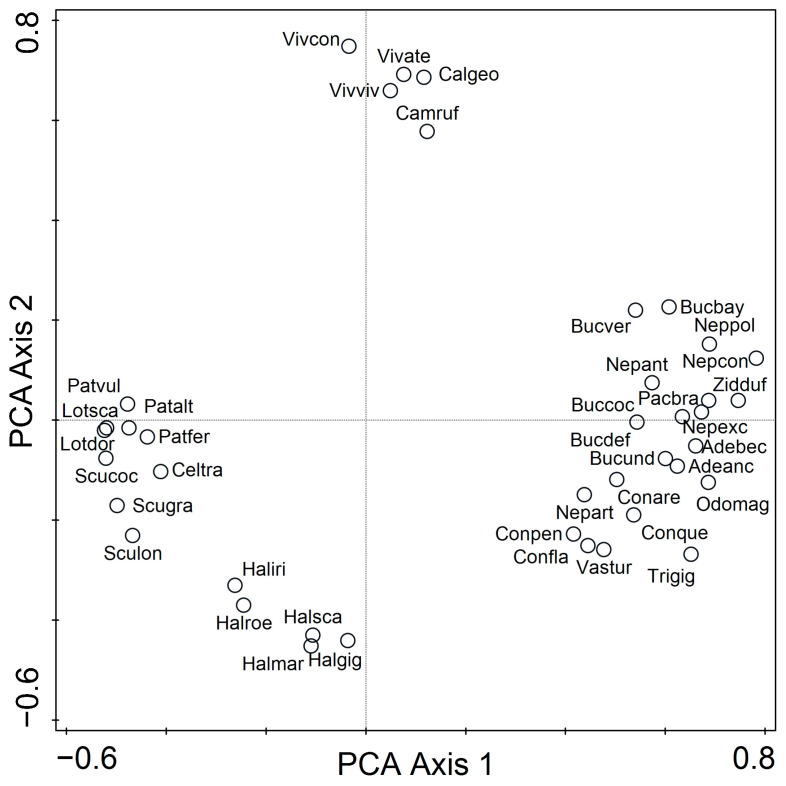
Principal Components Analysis (PCA) ordination diagram of 40 species selected based on best fit. Only the first two PCA axes are represented. Codes of species are represented by the first three letters of the genus followed by the first three letters of the species.

In the ordination space, the predictors with the best fit are widely dispersed (Figure 4), indicating a lack of correlation and of redundancy among them. The first axis is related to food richness and physical habitat characteristics, and the second axis to the freshwater-marine gradient. The 40 best-fitting species form three groups, separated along the first two axes (Figure 5). Along the first axis, the group of deep-water, large-sized marine species includes *Buccinum* spp., *Conus* spp., *Neptunea* spp. At the other end of this gradient, in tidal, food-rich, rocky environments, are *Patella* spp., *Scutellastra* spp., *Lottia* spp. and *Haliotis* spp. Along this first ordination axis, freshwater species have an intermediate position. However, they are well differentiated along the second axis and best represented by *Viviparus* spp., *Callinina georgiana* (I. Lea, 1834) and *Campeloma rufum* (Haldeman, 1841).

### 3.3. Responses of Longevity to Categories of Predictors

**Figure 6:**
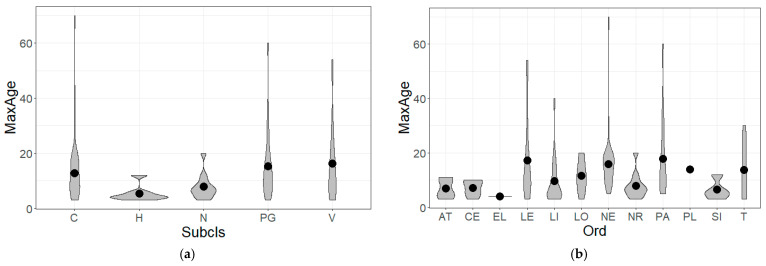
Violin plots for the probability distribution of longevity (MaxAge, in years) by (**a**) subclass (Subcls) and (**b**) order (Ord). Dots mark estimates of means based on GLM with gamma distribution. Codes of taxa are given in Table 1.

Because of the hierarchical nature of the taxonomic predictors, the effect of subclass and order could not be tested together. Maximal age was significantly predicted by the subclass (Subcls, χ^2^ = 18.9, df = 4, *p* < 0.001, explained deviance = 12.5%). The estimated mean maximal age was highest in Vetigastropoda (V), followed by Patellogastropoda (PG) and Caenogastropoda (C) and lowest in Heterobranchia (H), followed by Neritimorpha (N) (Figure 6a).

Longevity was significantly predicted by order (Ord) (χ^2^ = 40.8, df = 11, *p* < 0.001, explained deviance = 25%). It decreased successively as follows: Patelloidea (PA, the most long-lived), Lepetellida (LE), Neogastropoda (NE), Pleurotomariida (PL), Trochida (T), Lottioidea (LO), Littorinimorpha (LI), Neritoidea (NR), CE Cerithimorpha, Architaenioglossa (AT), Siphonariida (SI), and Ellobiida (EL, the shortest-lived) (Figure 6b).

**Figure 7:**
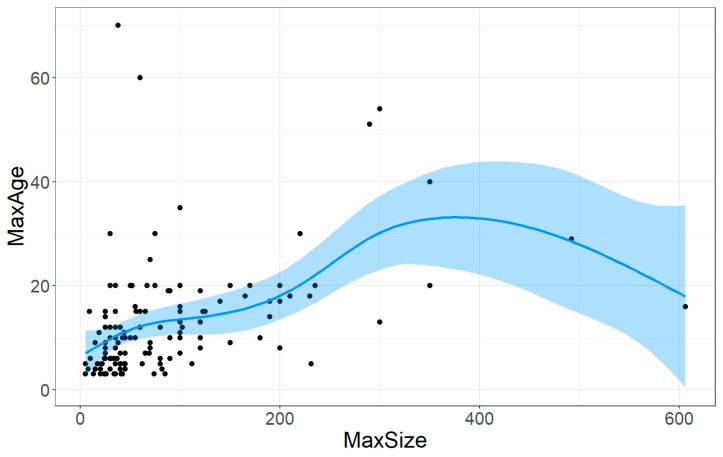
Effect of maximal size (MaxSize, mm) on longevity (MaxAge, years) in the generalised additive model (GAM). Light blue area represents the 95% confidence interval (*p* < 0.001, explained deviance = 22.9%).

Of the two morphological variables included in the analyses—maximal size (MaxSize) and shell thickness (ShellTh)—only size had a significant effect on lifespan. The GAM (χ^2^ = 34.4, df = 4.26, *p* < 0.001, explained deviance = 22.9%) illustrated the increase in longevity with maximal body size in small species and a decrease in large species, for which the degree of uncertainty is high (Figure 7), because of the small sample size of large gastropods.

**Figure A2:**
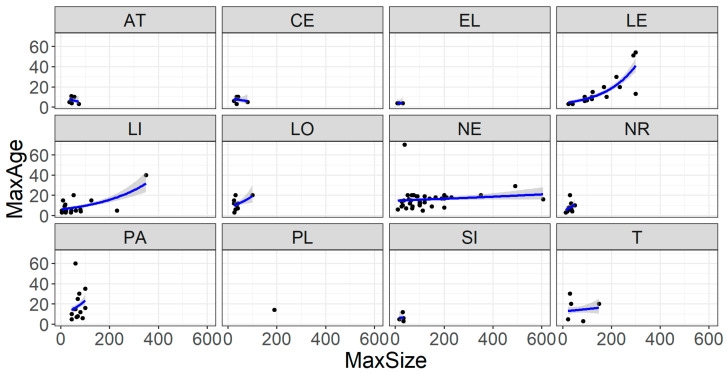
Relationships between longevity (MaxAge, years) and size (MaxSize, mm) separated by taxonomic group (order or superfamily, Ord; codes are given in Table 1).

When considering the differential responses among taxa by including taxon as a random factor, there was no effect of the subclass (χ^2^ = 2.1, df = 1, *p* = 0.147), but there were significant differences when the order was considered. The gamma GLMM with random intercepts and slopes for Ord (χ^2^ = 15.6, df = 2, *p* < 0.001) explained 48.7% of the variation in longevity, and most of this (29.5%) was accounted for by the differences among taxa (Figure A2).

**Figure 8:**
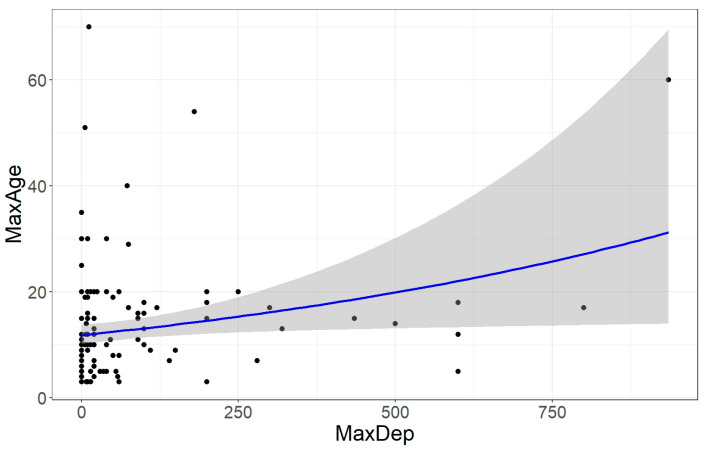
Effect of maximal depth (MaxDep, m) on longevity (MaxAge, years) in the generalised additive model (GAM). Shaded area represents the 95% confidence interval (*p* = 0.002, explained deviance = 6%).

Longevity varied in relation to depth (MaxDep and MinDep), but only the relation with MaxDep was significant (χ^2^ = 8.9, df = 1, *p* = 0.002, explained deviance = 6%). MaxAge increased slightly with maximal depth, but because of the limited data available, estimates of longevity for deep-sea species have a high degree of uncertainty (Figure 8).

**Figure 9:**
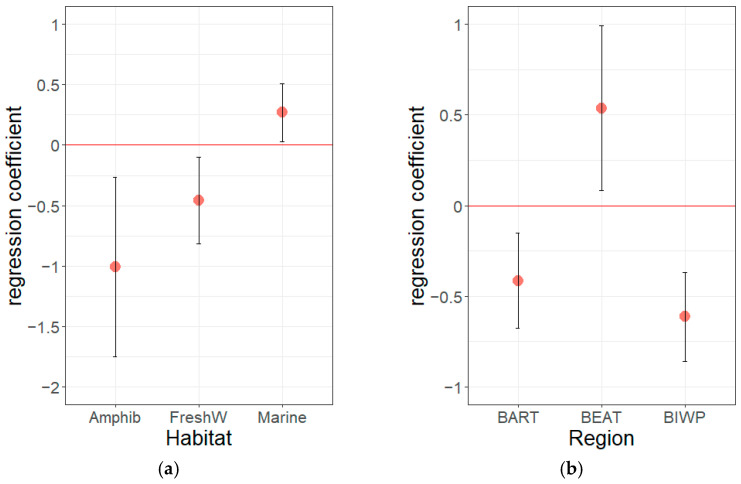
Regression coefficients of GLM with gamma distribution relating (MaxAge, in years) by the habitats (**a**) and biogeographical regions (**b**) included in the most parsimonious model: (**a**) marine (Marine), freshwater (FreshW), wetland (Amphib), (**b**) Arctic-temperate (BART), Eastern Atlantic (BEAT) and Indo-West Pacific (BIWP). Red dots mark the estimated regression coefficients and the error bars their 95% confidence intervals. The red horizontal line marks the lack of effect. Positive coefficients are for habitats or regions where mean longevity is higher than elsewhere and negative for lower.

The relationship with the habitat was significant (χ^2^ = 23.8, df = 6, *p* < 0.001, explained deviance = 15.4%). Longevity was highest in marine species and decreased in order with tidal zone (TidalZ, 1.5 times longer than elsewhere), above high tide level (AbTidZ), brackish water (BrackW), and freshwater (FreshW) being the shortest in amphibious gastropods (Amphib, 4.7 times shorter than aquatic species). A significant relation with salinity was shown, the longevity decreasing from marine (χ^2^ = 4.6, df = 1, *p* = 0.032) to freshwater habitats (χ^2^ = 5.4, df = 1, *p* = 0.020) and then towards wetland (χ^2^ = 5.1, df = 1, *p* = 0.023) environments (Figure 9a); these being the predictors that entered the most parsimonious model (χ^2^ = 21.7, df = 3, *p* < 0.001, explained deviance = 14.1%).

At a larger scale, at the boundary between marine and terrestrial environments, the effects of tidal zones were significant, with a weak relationship. However, only species living in the lower tidal zones showed a significant difference from species elsewhere (χ^2^ = 7.8, df = 1, *p* = 0.005, explained deviance = 5.3%), being longer-lived.

Considering the relations with the biogeographic regions, longevity was significantly explained by these (χ^2^ = 25.5, df = 6, *p* < 0.001, explained deviance = 16.4%). Maximal age decreased in the following order: BSOC (2.6 times longer than elsewhere), BEAT, BEPC, BWAT, BART, and the last being BIWP. Testing separately, BIWP (χ^2^ = 8.6, df = 1, *p* = 0.003), BEPC (χ^2^ = 7.6, df = 1, *p* = 0.005) and BSOC (χ^2^ = 6.2, df = 1, *p* = 0.012) had significant simple effects, but the most parsimonious model (χ^2^ = 20.5, df = 3, *p* < 0.001, explained deviance = 13.4%) included BIWP and BART, where gastropods are shorter-lived than elsewhere, and BEAT, where the estimated mean maximal age was largest (Figure 9b).

The relation to the climate was also significant but weak (χ^2^ = 8.7, df = 3, *p* = 0.033, explained deviance = 5.9%), hinting at the increase in longevity from warm towards arctic climate. However, only the effect of polar climate (CPOL) was significant (χ^2^ = 8.3, df = 1, *p* = 0.004, explained deviance = 5.7%). Tropical and temperate climates were characterised by similar snail longevity.

The relation with the trophic regime was significant (χ^2^ = 18.2, df = 6, *p* = 0.005, explained deviance = 12%) and somewhat comparable with the extent of the effect of habitat. Longevity decreased in the following order: omnivores (Omni), scavengers (Scav), herbivores (Herb), predators (Pred), detritivores (Detr), and filter-feeders (Filt). Among the diet types, only detritivores (χ^2^ = 6.8, df = 1, *p* = 0.008) and filter-feeders (χ^2^ = 4.2, df = 1, *p* = 0.041) had a significantly negative effect on the maximal age (χ^2^ = 14.1, df = 2, *p* < 0.001, explained deviance = 9.8%).

Aggregation had only a marginally significant effect (χ^2^ = 2.8, df = 1, *p* = 0.091) on longevity, with aggregation decreasing maximal age.

Although longevity was slightly longer in habitats with higher biodiversity, the relationship between maximal age and the biotope species richness was not significant. Food availability in the habitat also did not affect maximal age.

The reproduction strategy had a significant effect on lifespan. Iteroparous species had, on average, 3.3 times longer life than semelparous species (χ^2^ = 24.7, df = 1, *p* < 0.001, explained deviance = 15.9%). The reproduction strategy was second only to maximal size in explaining longevity.

Endemic species have slightly shorter life (χ^2^ = 4.7, df = 1, *p* = 0.030, explained deviance = 4.9%). Species with continuous range live slightly longer than those with fragmented area distribution (χ^2^ = 6.9, *p* = 0.008, explained deviance = 5%).

Habitat stability had a significant but weak positive effect on maximal age (χ^2^ = 4.2, df = 1, *p* = 0.041, explained deviance = 2.8%).

Overall, the type of substratum on which the species lives (e.g., mud, clay, sand, rocks) had a slight effect on longevity (χ^2^ = 17.5, df = 9, *p* = 0.041, explained deviance = 11.6%). However, among all substrates, only mangroves (HMang) had a significant simple effect (χ^2^ = 5.4, *p* = 0.020, explained deviance = 3.7%).

The relative dimension of the range, whether the species is invasive or not, and predation did not show a significant effect on longevity for our dataset including species with a lifespan exceeding three years.

The best models relating longevity to life history, environment and biogeographical characteristics were GAMs with a gamma distribution. The set of competing models included the smooth terms of MaxSize (maximal size) and MaxDep (maximal depth), Repr (reproduction), Detr (detritivorous), Amphib (amphibious), TidalZ (tidal zone), BSOC (Southern Ocean), BEPC (Eastern Pacific), BIWP (Indo-West Pacific), HMang (mangroves), HSalt (salt marshland), and HGrave (gravel) (Table 2).

**Figure 10:**
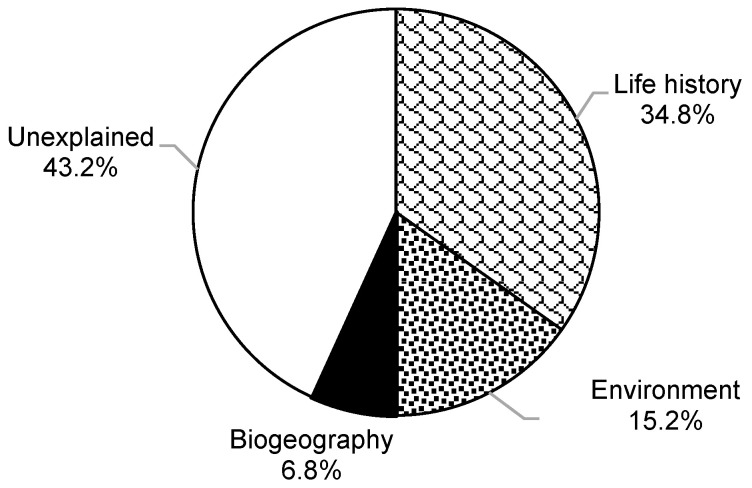
Partitioning of deviance among the groups of predictors in the best GAM explaining gastropod longevity. Life history traits include MaxSize (maximum size), Repr (reproduction strategy) and Detr (detritivorous), environment includes Amphib (amphibious), TidalZ (tidal zone), MaxDep (maximum depth), HSalt (salty marshland), HMang (mangroves) and HGrave (gravel), and biogeography includes BSOC (Southern Ocean), BIWP (Indo-West Pacific) and BEPC (Eastern Pacific) geographic ranges.

The average deviance explained by the set of best models was 56.8%. Of this, the life history traits (in a broad sense, i.e., MaxSize, Repr, Detr) explained the largest part (34.8%), followed by environment, represented by habitat (TidalZ, Amphib, MaxDep) and type of substrate (HSalt, HMang, HGrave) (15.2%) and biogeography (BSOC, BIWP, BEPC—6.8%) (Table 2, Figure 10). On a variable-based effect, biogeography had similar importance as environment, but none of the biogeographical variables was included in all the competing models, in contrast to MaxSize, Repr, TidalZ, Amphib and HSalt (Table 2).

**Figure 11:**
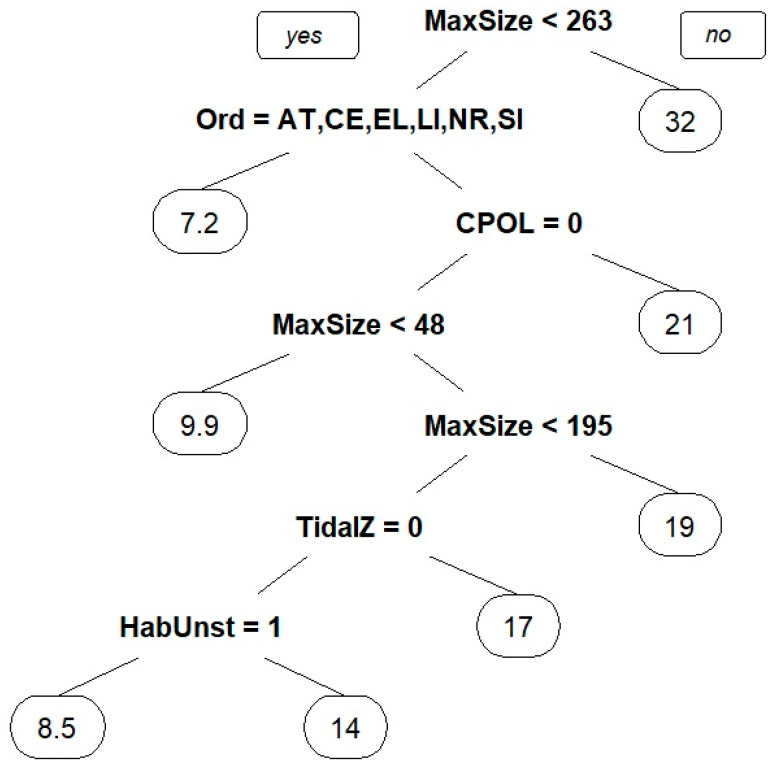
Regression tree between longevity and the significant predictors. Branching is done according to the rule of true (yes—left) versus false (no—right). Circled numbers denote the expected lifespan of gastropods with the particular combination of traits. Variable codes are given in Table 1.

The regression tree reiterates the importance of body size (MaxSize) for longevity, both on its own and in combination with other predictors, making it a recurrent predictor in our model (Figure 11). In contrast to regression models, where the effect of taxa is obscured by other predictors, the regression tree can use order (Ord) to predict the maximum age. Orders Architaenioglossa (AT), Cerithimorpha (CE), Ellobiida (EL), Littorinimorpha (LI), Neritoidea (NR) and Siphonariida (SI) form a distinct group of small-sized and short-lived gastropods. Polar climate (CPOL), tidal zone (TidalZ) and habitat stability (HabUnst) were also shown to predict longevity in a hierarchical manner, with cold climate and intertidal gastropods living in stable habitats having a longer expected lifespan.

### 3.4. Model Robustness

Using alternative lifespan cutoffs, we obtained consistent results (Table 3—GLM). For Repr and Amphib, we could not construct all models because long-lived species are all iteroparous and none is amphibious.

In contrast, in the comparative phylogenetic regression models, there were some differences, with most of the predictors decreasing their effect. However, for the most important predictors in GLM—MaxSize, Repr, MaxDep, Marine and the biogeographical regions BIWP, BEPC and BSOC—the effect remained significant or marginally significant (Table 3—PGEE). In most models, Pagel’s λ ranged between 0.32 and 0.44, indicating a moderate amount of phylogenetic covariance in the residuals after accounting for the effects of the predictors. Only for MaxSize was the residual covariance much closer to independent (λ = 0.18). Similarly, in the phylogenetic autoregression models, phylogeny accounted for only 12.5% of the variation in the maximal age, and Moran’s I showed a non-significant autocorrelation across the phylogeny (*p* = 0.408).

## 4. Discussion

Longevity represents a fundamental life history trait shaped by ecological, physiological and evolutionary constraints. Despite extensive research in vertebrates, the drivers of maximum lifespan in aquatic invertebrates remain insufficiently understood. We analysed a comprehensive dataset of 133 species, encompassing morphological, ecological, trophic, habitat and environmental variables, to identify predictors of maximum lifespan (MaxAge). Using generalised linear models with gamma-log distribution, generalised additive models, random forest regression, and regression tree analysis, we evaluated the relative contributions of body size, habitat characteristics, trophic strategy, salinity regime, depth distribution and environmental stability.

As already pointed out in the introduction, there are numerous data on the lifespan of aquatic gastropods, including the cases of relative longevity, but, contrary to the much better-studied Bivalvia, little has been said about the possible background of unusually long lifespan in the Gastropoda. In the present study, we examined some possible associations between maximum lifespan and known factors at the specimen and population levels. Despite the rather poor state of knowledge of the biology and ecology of these animals, some reliable conclusions about the factors affecting maximum lifespan can be drawn and, in our opinion, may enrich our understanding of this aspect of gastropod ontogeny and evolution.

Species combining large size, iteroparity, and occupation of environmentally stable marine habitats were found to form the longest-lived group. In contrast, small-bodied, amphibious, and semelparous species formed the shortest-lived cluster.

Abele and Philipp [30], discussing longevity in the Bivalvia, formulated “Principle 1: The Bivalve Shell—Protection from Predation and a Private-Protective Niche for Physicochemical Control and Self-Induced Depression of Metabolic Rate”. They stressed that the ecological stress theory of ageing and hormesis predicts that critical fluctuations in physicochemical factors are major drivers of cellular ageing. Such a private-protective niche also characterises gastropods, especially those bearing a fully developed operculum. Heller [47] stressed that all the gastropods with reduced shells are short-lived, although terrestrial *Testacella* spp. live up to six years [82]. All the marine and freshwater snails living more than three years and considered in our study have shells, and all these shells are neither reduced nor thin, translucent, or fragile. However, shell thickness (thin, thick or massive) did not have a significant effect on the maximum age.

We have found that reproductive strategy has a very strong effect on longevity. Iteroparous gastropods (K-strategists) live 3.3 times longer than semelparous (r-strategists) ones. Both Calow [83] and Geraerts and Joosse [84] reported that freshwater snails with an iteroparous strategy, all of them not representatives of Heterobranchia, inhabit small, closed water bodies, where there is more competition, more density-dependent control, and hence a greater premium on the survival of a large, “experienced” adult. However, their opinion is confirmed neither by the ecology of long-lived freshwater gastropods nor by speculative claims about competition and density-dependent control supposedly higher in small, closed water bodies. Iteroparous K-strategists are often thought to invest in parental care, but among the nearly longest-living representatives of *Haliotis*, the free-swimming larva emerges already at the trochophore phase, similar to long-lived Patellogastropoda and Neritopsina. There is no true viviparity (with feeding of the developing embryo) in the non-terrestrial gastropods, but ovoviviparity can be found in several cases in Caenogastropoda: among long-lived in Architaenioglossa (freshwater Viviparidae), as well as in Littorinimorpha: *Littorina saxatilis* (Olivi, 1792) and *Melarhaphe neritoides* (Linnaeus, 1758), but their lifespan (11 and 15 years, respectively) is even shorter than in oviparous *Littorina littorea* (Linnaeus, 1758) (20 years). Ovoviviparous *Buccinum undatum* Linnaeus, 1758, does not live longer than oviparous Buccinidae. Apart from ovoviviparity [85,86], gastropods present a large variety of parental care adaptations. Some of these adaptations include the enclosure of unfertilised eggs (nurse eggs) within egg capsules, the incubation of the egg capsules under the foot, the preparation of surfaces for capsule deposition, the attachment of egg capsules on the parental shell [85,86] and the storage of eggs in the mantle cavity [87,88].

We have found that longevity increased exponentially with size up to about 300 mm, peaked between 350 and 400 mm, and decreased at larger sizes. This confirms a general rule—strong allometric scaling—that larger animals are longer-lived (e.g., [5]; an extreme lifespan may emerge from indirect selection on a co-varying trait). One potential explanation for the decrease in longevity at large sizes might be differences in the patterns of this relationship among gastropod phylogenetic lineages. The only order including really large species, Neogastropoda, is characterised by the absence of the positive relationship between longevity and size seen in Lepetelloidea, Littorinoidea, Patelloidea or Neritopsina. It is not the only order in which longevity does not respond to size, but it is probably the one driving the overall downward trend shown by the GAM. Another potential explanation is the small sample size of large snails, leading to a very high degree of uncertainty concerning the effect of size on longevity for the upper interval of variation in body size. The phylogenetic comparative model confirmed the strong body size-lifespan relationship and the low Pagel’s λ, indicating that the residual variation around this relationship exhibited only weak phylogenetic dependence.

Abele and Philipp [30] presented for the long-lived Bivalvia “Principle 3: Slow Growth, Sex Change and Reproduction to Life’s End”. They stressed the absence of reproductive senility, which means constant fertility and reproductive output independent of age, as characteristic of long-lived Bivalvia. Also, in aquatic long-lived Gastropoda, maturation often begins late, especially in females, and adults exhibit asymptotic, slow growth and prolonged reproduction (spawning periods) throughout the year, rather than a distinctly timed spawning peak. This strategy minimises stress from exhausting reproduction in early life and reduces oxidative damage accumulation during the extended reproductive lifespan, but it may be even more sophisticated [30]. Many long-lived dioecious bivalves and some long-lived gastropods (such as *Patella ferruginea* Gmelin, 1791, 35 years) are protandric (e.g., [89]); old big specimens produce more eggs. For example, in *Aliger gigas*, the number of produced eggs increases with female size and age (correlation with lip thickness: [90]) and bigger females of *Haliotis kamtschatkana* Jonas, 1845, produce more eggs [91]. However, the general pattern seems more complicated: in *Haliotis rufescens*, fecundity, measured as the estimated number of mature eggs per female, increased exponentially with shell length until peaking at 215 mm, after which it began to decline. The largest studied female, with a 260 mm long shell, had over 80% necrotic eggs [92], which clearly reflects reproductive senility. On the other hand, [93] reported that in *H. rufescens*, both fertility and mortality were stable throughout life.

Protandry in *Patella ferruginea* results in twice as many females as males [94]. The sex ratio, in general, varies between the long-lived gastropods. For example, in *Aliger gigas* the sex ratio is 1:1 [45] and is similar in several species of *Haliotis* [95], but in *Haliotis gigantea* Gmelin, 1792, the female:male ratio is 1:1.7 [96] and thus, males dominate. In *Nacella concinna*, whose lifespan approaches 50 years, the sex ratio is 1.4 females:1 male [97]. The sex ratio in *Adelomelon ancilla* ([Lightfoot], 1786) is about 1:1 [98], but there are twice as many females in *A. beckii* (Broderip, 1836) [99].

While the increased probability of mortality with age has been considered a universal trait among organisms, recent comparative studies of tortoises [100] and nonavian reptiles and amphibians [101,102] have identified several cases in which ageing rates are negligibly low, or even negative. Data on the gastropods studied are scarce, but the picture for at least some species appears similar (e.g., [93]).

Our results illustrate the importance of phylogeny; subclass and order or superfamily placement may predict maximal age. However, in the multiple regression models, taxa (Subcls and Ord) had no significant effect in addition to the considered predictors, neither as factors, nor as separate levels (when included as binary predictors), which indicates that the variations in longevity among taxa may be explained by their morphological, ecological and geographical characteristics. In contrast to multiple regression models, where the effect of taxa is obscured by other predictors, in regression trees, they can be used to predict the maximal age. We did not confirm the opinion that in a genus there are either short-lived or long-lived species [47]. Within a genus, there is often a wide variability, such as *Cellana*: 5–15 years; *Scutellastra*: 8–25 years; *Lottia*: 6–20 years; *Patelloida* and *Fissurella*: 3–10 years; *Haliotis*: 7–54 years; *Nerita*: 4–12 years; and *Siphonaria*: 3–12 years.

In ectothermic aquatic invertebrates, metabolic rate is strongly modulated by environmental temperature and water depth, linking ecological conditions directly to mitochondrial function. Species inhabiting colder or more stable environments may experience reduced oxidative damage and slower metabolic turnover, contributing to extended lifespan. Our findings that body size and habitat stability predict longevity are consistent with mitochondrial [6] and metabolic theories of ageing, suggesting that ecological context shapes lifespan through bioenergetic mechanisms.

Maximum depth below the tidal zone was positively related to maximum age, although the data were scarce. This can be explained by a more stable environment and lower predation intensity, but also food shortage—caloric restrictions, resulting in slower metabolic rates [103]. As concerns feeding, longevity decreased in order: omnivorous—scavenger—herbivorous—predator—detritivore—filter feeder. The significant negative effect was found only for detritivores. Detritus is not only low in calories but also low in certain elements. The most opportunistic omnivory was associated with the longest lifespan. The most caloric food of predators did not result in higher longevity, which proves again that caloric restrictions prolong lifespan.

We found that species inhabiting tidal zones live on average 1.5 times longer than those living in other environments. This may contradict the ecological stress theory of ageing and hormesis [30], which predicts that critical fluctuations in physicochemical factors are major drivers of cellular ageing. Despite the highly variable conditions in this zone, the astronomically driven rhythm of tides remains regular and predictable. We also have to consider the overrepresentation of the species inhabiting the tidal zone in our data set, since this environment is most easily accessible for researchers. Amphibiotic species, whose habitat is more unpredictable, live 4.7 times shorter than the aquatic ones.

Among the geographical distributions, the Southern Ocean (with unique cold-adapted species) is characterised by long-lived species, with an average maximal age 2.6 times higher than in other geographic areas. This phenomenon was observed for several animals, especially for Bivalvia [32,104,105,106]. In our study, the most parsimonious model included Indo-West Pacific and Arctic-temperate regions, where gastropods are shorter-lived than elsewhere, as well as the Eastern Atlantic. The reduced lifespan in the Indo-West Pacific is rather unexpected, given that the Indo-West Pacific molluscan fauna is the richest worldwide (e.g., [107]). On the other hand, in the Eastern Atlantic, although long-lived species were absent, the estimated mean maximum age was higher than in any other region. Perhaps these differences between the biogeographic regions are biassed by the evidently uneven sampling across them.

Powell and Cummins [42] suggested the association of the maximum lifespan in the marine gastropods with long-term cycles in the benthic communities, caused by periodic fluctuations in environmental conditions, but our data did not confirm such relationships; violin diagrams for both subclasses and orders or superfamilies do not show such peaks of longevity.

According to Chen and Maklakov [108], a shorter lifespan evolves under high random mortality, as classically assumed, whereas high condition-dependent mortality leads to a longer lifespan, supporting a key role of condition dependence in the evolution of ageing. In our dataset, the random forest regression indicated that higher overall predation may, in some cases, lead to a longer lifespan, as suggested by Abrams [109]. Unfortunately, the knowledge on the interspecific relationships in our selection of species, spanning across a wide range of taxa from very different environments and exhibiting diverse life history strategies, is sparse. When not available, to make comparisons possible, we used some estimates, taking into account the abundance of predators and the vulnerability of the snail (considering size, shell thickness, behaviour, microhabitat, etc.). The lack of significance of predation as a predictor of lifespan in the regression models probably reflects the scantiness of reliable knowledge on this aspect.

The convergence among parametric (GLM and GAM), comparative phylogenetic regression (PGLS) and machine learning approaches (RT and RF) strengthens confidence that body size and life history strategy represent the primary axes structuring lifespan variation, and their effect is not dependent on species selection or phylogenetic clustering. Random forest regression confirmed that maximal size and reproduction strategy are the primary predictors of longevity. Habitat-related variables (tidal zone, amphibious, salty marsh) and depth also ranked highly in variable importance based on permutation (%IncMSE).

Regression tree analysis identified maximum shell size as the first and most influential split, confirming its central role in driving, or at least predicting, lifespan. Subsequent splits involved taxonomy, climate and habitat-related variables, revealing interaction structures among predictors. The tree predicts longevity hierarchically and highlights threshold effects and combinations of ecological strategies that are not readily captured by regression models.

The PCA ordination diagram illustrates a phylogenetic signal in the species’ traits, despite variation in maximum age among congeneric species and the lack of a significant phylogenetic effect revealed by Moran’s I autocorrelation.

To elucidate the underlying mechanisms of longevity and test mechanistic ageing hypotheses, additional response variables should also be considered (such as growth rates and age at maturity) in relation to a wider range of potential drivers, including physiological traits such as metabolic rates, oxygen consumption and oxidative stress biomarkers. A more balanced dataset, containing a larger sample of large-sized and deep-sea gastropod species, would decrease model uncertainty and improve the interpretation of the results. In addition, including new categories of snails, such as tropical deep-sea and extreme-environment (e.g., hydrothermal vents) taxa, would improve model generality.

## 5. Conclusions

Longevity is shaped by life history trade-offs between growth, reproduction and maintenance. In ectotherms, metabolic constraints, environmental stability and predation pressure jointly influence survival strategies. Our analyses demonstrate that body size, habitat stability, salinity regime and trophic mode are consistent predictors of maximum lifespan across gastropod taxa. These findings support life history theory and metabolic scaling predictions, indicating that species with slower growth, larger body size and occupation of environmentally stable habitats exhibit extended longevity. The convergence of parametric (GLM and GAM), comparative phylogenetic regression (PGLS) and machine learning (RF and RT) approaches strengthens confidence that body size, reproductive strategy and environmental stability represent the primary axes structuring lifespan variation. Our models could be used to predict how longevity distributions would change in response to rising water temperatures and to test whether shallow-water species would experience reduced lifespans.

## Figures and Tables

**Table 1:** The variables used in the data analysis. For binary variables, where not specified otherwise, 0 is for No and 1 for Yes.

Category	Code	Definition	Type
Response variable	MaxAge	maximum age (longevity in years)	numerical
Explanatory variables (predictors)			
Phylogeny	Subcl	subclass: PG—Patellogastropoda, V—Vetigastropoda, N—Neritimorpha, C—Caenogastropoda, H—Heterobranchia	factor
	Ord	order or superfamily: LO—Lottioidea, PA—Patelloidea, LE—Lepetellida, PL—Pleurotomariida, T—Trochida, NR—Neritoidea, AT—Architaenioglossa, LI—Littorinimorpha, NE—Neogastropoda, CE—Cerithimorpha (incertae sedis), SI—Siphonariida, EL—Ellobiida	factor
Morphology	MaxSize	maximum size (mm)	numerical
	ShellTh	shell thickness: 0—thin, 1—thick, 2—massive	ordinal
Reproductive strategy	Repr	reproduction: 0—semelparous; 1—iteroparous	binary
Trophic regime	Herb	herbivorous	binary
	Detr	detritophagous	binary
	Filt	filter-feeder	binary
	Pred	predator	binary
	Scav	scavenger	binary
	Omni	omnivore	binary
Predation pressure	IntPred	overall intensity of predation: 0–weak, 1–medium, 2–strong	ordinal
	JuvPred	predation is more intense on young specimens	binary
Aggregation	Aggreg	aggregation	binary
Habitat	Amphib	amphibiotic	binary
	Marine	marine (below tide zone)	binary
	TidalZ	tidal zone	binary
	AbTidZ	above high tide level	binary
	BrackW	brackish water	binary
	FreshW	freshwater	binary
Habitat characteristics	BioRich	biotope rich in species	binary
	BioPoor	biotope poor in species	binary
	FoodRich	food resources rich	binary
	FoodPoor	food resources limited	binary
	HabStab	stable habitat	binary
	HabUnst	unstable habitat	binary
Depth	MinDep	minimum depth (m)	numerical
	MaxDep	maximum depth below low water level (m)	numerical
	UpTZ	in upper tide zone	binary
	LowTZ	in lower tide zone	binary
Biogeography	BIWP	Indo-West Pacific (Indian Ocean and the western Pacific)	binary
	BEPC	Eastern Pacific (western coasts of North and South America)	binary
	BWAT	Western Atlantic (including Caribbean Sea and Gulf of Mexico)	binary
	BEAT	Eastern Atlantic	binary
	BSOC	Southern Ocean (with unique cold-adapted species)	binary
	BART	Arctic-temperate (N Pacific, N Atlantic, Mediterranean)	binary
Climate	CPOL	polar	binary
	CTEM	temperate	binary
	CTRO	tropical	binary
Range	RangVR	range very restricted	binary
	RangR	restricted range	binary
	RangW	wide range	binary
	RangVW	very wide range	binary
	ContRange	continuous range	binary
	End	endemic	binary
	Inv	invasive	binary
Substratum	HRock	rocks	binary
	HCora	corals	binary
	HMang	mangroves	binary
	HSand	sand	binary
	HMud	mud	binary
	HGrave	gravel	binary
	HMacr	macrophytes	binary
	HClay	clay	binary
	HSalt	salt marshlands	binary

**Table 2:** Best competing gamma GAMs (M1 to M8) relating longevity (MaxAge) to life history, environment and biogeography variables. AICc—Akaike Information Criterion corrected for small samples; Delta AICc—difference between the AICc of the best model and the evaluated model; Expl.dev—deviance explained by the model—used as a measure of the variation in the response variable explained by the predictors; s(MaxSize) and s(MaxDep)—nonlinear smooth effects of maximum shell size and maximum depth. For these there are no regression coefficients, because size and depth have a nonlinear effect; thus, the regression coefficient is not constant. For averaged regression coefficients the 95% confidence intervals are given in parentheses. Codes of variables are given in Table 1.

Predictor	M1	M2	M3	M4	M5	M6	M7	M8	Averaged Coefficient
Life history	Averaged Expl.dev = 34.8%								
s(MaxSize)	+	+	+	+	+	+	+	+	
Repr	+	+	+	+	+	+	+	+	1.08 (0.72, 1.45)
Detr				+					−0.11 (−0.34, 0.11)
Environment	Averaged Expl.dev = 15.2%								
Amphib	+	+	+	+	+	+	+	+	−1.62 (−2.28, −0.96)
TidalZ	+	+	+	+	+	+	+	+	0.38 (0.18, 0.59)
s(MaxDep)						+			
HSalt	+	+	+	+	+	+	+	+	0.83 (0.37, 1.28)
HGrave	+	+	+	+	+	+	+		−0.22 (−0.44, −0.01)
HMang	+	+	+	+	+	+		+	−0.44 (−0.82, 0.06)
Biogeography	Averaged Expl.dev = 6.8%								
BSOC	+	+	+	+	+		+	+	1.1 (0.36, 1.84)
BIWP	+			+	+	+	+		−0.17 (−0.37, 0.02)
BEPC		+			+			+	0.22 (−0.08, 0.52)
Model performance									
AICc	819.3	820.1	820.2	820.8	820.8	821.0	821.0	821.2	
Delta AICc	0	0.79	0.95	1.47	1.48	1.68	1.74	1.94	
Model weight	0.223	0.150	0.138	0.107	0.106	0.096	0.093	0.084	
Explained deviance (%)	57.3	56.4	56	57.8	57.2	58.2	55.7	54.9	56.8

**Table 3:** Significance and coefficient estimates of predictors in the sensitivity analysis models explaining lifespan. GLMs—Generalised linear models with gamma distribution using alternative lifespan cutoffs: M3—three, M4—four, M5—five and M6—six years; PGLS—Phylogenetic Generalised Least Squares; lambda—Pagel’s λ. Significant effects are in bold. Only predictors with significant effects are included. Predictors with consistent effects (*p* < 0.1) across all models are in bold. Predictor codes are explained in Table 1.

Predictor	GLM								PGLS		
	*p*-Value				Coefficient						
	M3	M4	M5	M6	M3	M4	M5	M6	Lambda	*p*-Value	Coeff
Subcls	**0.001**	**0.000**	**0.023**	**0.011**	-	-	-	-	-	-	-
Ord	**0.000**	**0.000**	**0.006**	**0.010**	-	-	-	-	-	-	-
Amphib	**0.008**	**0.021**	-	-	**−1.278**	**−1.253**	-	-	0.41	0.200	−0.616
**Marine**	**0.001**	**0.005**	**0.016**	**0.026**	**0.398**	**0.327**	**0.281**	**0.256**	**0.32**	**0.061**	**0.278**
FreshW	**0.001**	**0.010**	**0.011**	**0.013**	**−0.657**	**−0.529**	**−0.524**	**−0.515**	0.40	0.158	−0.319
**MaxSize**	**0.000**	**0.000**	**0.000**	**0.000**	**0.003**	**0.003**	**0.002**	**0.002**	**0.18**	**0.000**	**0.003**
LowTZ	**0.005**	**0.008**	**0.022**	**0.018**	**0.339**	**0.314**	**0.267**	**0.270**	0.36	0.214	0.185
**MaxDep**	**0.003**	**0.005**	**0.009**	**0.009**	**0.001**	**0.001**	**0.001**	**0.001**	**0.34**	**0.012**	**0.001**
Detr	**0.001**	**0.008**	**0.031**	0.143	**−0.448**	**−0.365**	**−0.299**	−0.209	0.42	0.727	−0.056
Filt	**0.005**	0.073	0.144	0.215	**−0.781**	−0.566	−0.487	−0.433	0.39	0.140	−0.431
**BIWP**	**0.003**	**0.004**	**0.001**	**0.001**	**−0.355**	**−0.334**	**−0.366**	**−0.364**	**0.43**	**0.016**	**−0.302**
**BEPC**	**0.006**	**0.013**	**0.023**	**0.035**	**0.488**	**0.413**	**0.365**	**0.325**	**0.44**	**0.016**	**0.457**
**BSOC**	**0.013**	**0.013**	**0.014**	**0.017**	**1.022**	**0.954**	**0.911**	**0.822**	**0.44**	**0.089**	**0.844**
CPOL	**0.004**	**0.008**	**0.014**	**0.017**	**0.537**	**0.463**	**0.416**	**0.388**	0.39	0.186	0.289
BioRich	**0.044**	0.067	0.118	0.059	**0.241**	0.212	0.178	0.210	0.42	0.605	0.076
HabStab	**0.041**	**0.045**	0.075	**0.026**	**0.253**	**0.240**	0.211	**0.256**	0.40	0.138	0.216
**Repr**	**0.000**	**0.050**	**0.034**	**-**	**1.376**	**1.029**	**1.073**	**-**	**0.32**	**0.000**	**1.073**
End	**0.031**	**0.043**	**0.088**	**0.033**	**−0.686**	**−0.646**	**−0.576**	**−0.668**	0.43	0.607	−0.155
ContRange	**0.007**	**0.006**	**0.006**	**0.003**	**0.394**	**0.388**	**0.384**	**0.410**	0.44	0.109	0.246
HMang	**0.020**	**0.005**	**0.007**	**0.014**	**−0.599**	**−0.672**	**−0.657**	**−0.627**	0.44	0.689	−0.096
Hmud	0.078	0.056	0.054	**0.022**	0.275	0.288	0.285	**0.333**	0.44	0.258	0.196

## Data Availability

All the data supporting reported results are publicly available in the Figshare data repository: https://doi.org/10.6084/m9.figshare.33103952.

## Abbreviations

Abbreviations of variables are given in Table 1. Abbreviations of species are represented by the first three letters of the genus followed by the first three letters of the species. The following abbreviations are used in this manuscript.

GLM	Generalised linear model
GLMM	Generalised linear mixed model
GAM	Generalised additive model
RF	Random forest
RT	Regression tree
PCA	Principal components analysis
%IncMSE	Percent Increase in Mean Squared Error

## Appendix A

**Table A1:** Simple effects of the variables considered as potential drivers of longevity in aquatic and semiaquatic gastropods. AIC—Akaike Information Criterion, none—null model including only the intercept. All models have one degree of freedom. Predictors with significant effects are in bold. Results are from generalised linear models with Gamma distribution. Codes of predictors are given in Table 1.

Variable	Deviance	AIC	Scaled Deviance	Coefficient	*p*
none	68.8	911.1	-	-	-
**Amphib**	**65.5**	**908.4**	**4.64**	**−1.282**	**0.031**
**Marine**	**63.7**	**906.0**	**7.11**	**0.403**	**0.008**
TidalZ	68.8	913.0	0.04	0.021	0.835
AbTidZ	68.8	913.1	0.00	0.000	0.974
BrackW	67.9	911.9	1.20	−0.212	0.273
**FreshW**	**63.6**	**905.8**	**7.22**	**−0.661**	**0.007**
**MaxSize**	**57.0**	**896.6**	**16.51**	**0.003**	**<0.001**
MinDep	68.6	912.8	0.27	0.002	0.603
UpTZ	68.0	911.9	1.16	0.162	0.281
**LowTZ**	**65.1**	**907.9**	**5.12**	**0.344**	**0.024**
**MaxDep**	**64.6**	**907.2**	**5.83**	**0.001**	**0.016**
Herb	68.7	912.9	0.12	−0.043	0.731
**Detr**	**63.9**	**906.2**	**6.87**	**−0.453**	**0.009**
**Filt**	**65.1**	**907.9**	**5.17**	**−0.786**	**0.023**
Pred	68.7	913.0	0.10	0.041	0.754
Scav	68.4	912.5	0.60	0.161	0.437
Omni	68.8	913.0	0.05	0.060	0.832
ShellTh	68.2	912.2	0.84	0.097	0.360
**BIWP**	**64.8**	**907.4**	**5.66**	**−0.360**	**0.017**
**BEPC**	**65.2**	**908.0**	**5.03**	**0.492**	**0.025**
BWAT	68.6	912.8	0.27	0.091	0.606
BEAT	68.1	912.1	0.91	0.294	0.339
**BSOC**	**65.9**	**908.9**	**4.11**	**1.017**	**0.043**
BART	68.5	912.6	0.45	−0.114	0.504
**CPOL**	**64.9**	**907.6**	**5.48**	**0.531**	**0.019**
CTEM	68.8	913.0	0.04	0.040	0.845
CTRO	68.8	913.0	0.02	−0.012	0.883
Aggreg	67.4	911.2	1.91	−0.211	0.167
BioRich	66.9	910.4	2.70	0.248	0.100
BioPoor	67.7	911.6	1.50	−0.196	0.220
FoodRich	68.5	912.7	0.39	−0.105	0.532
FoodPoor	68.4	912.5	0.55	0.110	0.458
HabStab	66.8	910.3	2.77	0.259	0.096
HabUnst	68.6	912.8	0.21	−0.083	0.643
**Repr**	**57.8**	**897.7**	**15.38**	**1.380**	**0.000**
RangVR	68.7	912.9	0.12	0.060	0.725
RangR	68.5	912.6	0.43	0.096	0.513
RangW	68.4	912.5	0.60	−0.174	0.440
RangVW	67.1	910.7	2.32	−0.544	0.128
End	66.6	910.0	3.10	−0.691	0.078
Inv	68.4	912.6	0.49	0.194	0.482
**ContRange**	**65.4**	**908.3**	**4.80**	**0.398**	**0.028**
HRock	68.8	913.0	0.03	−0.021	0.857
Hcora	68.1	912.0	1.02	−0.240	0.312
HMang	66.2	909.5	3.57	−0.604	0.059
Hsand	68.3	912.4	0.70	0.125	0.401
Hmud	67.3	911.0	2.07	0.269	0.150
HGrave	68.8	913.0	0.02	−0.031	0.884
HMacr	68.2	912.2	0.87	0.235	0.350
HClay	67.9	911.8	1.30	−0.224	0.254
HSalt	68.6	912.8	0.24	0.152	0.624
IntPred	68.6	912.9	0.20	−0.068	0.652
JuvPred	68.7	913.0	0.10	0.037	0.751
